# Iterative Network for Disparity Prediction with Infrared and Visible Light Images Based on Common Features

**DOI:** 10.3390/s24010196

**Published:** 2023-12-28

**Authors:** Ziang Zhang, Li Li, Weiqi Jin, Zanxi Qu

**Affiliations:** MOE Key Laboratory of Optoelectronic Imaging Technology and System, Beijing Institute of Technology, Beijing 100081, China; 3120210519@bit.edu.cn (Z.Z.); jinwq@bit.edu.cn (W.J.); qu425277261@163.com (Z.Q.)

**Keywords:** binocular stereo vision, disparity prediction, common features, multiband imaging

## Abstract

In recent years, the range of applications that utilize multiband imaging has significantly expanded. However, it is difficult to utilize multichannel heterogeneous images to achieve a spectral complementarity advantage and obtain accurate depth prediction based on traditional systems. In this study, we investigate CFNet, an iterative prediction network, for disparity prediction with infrared and visible light images based on common features. CFNet consists of several components, including a common feature extraction subnetwork, context subnetwork, multimodal information acquisition subnetwork, and a cascaded convolutional gated recurrent subnetwork. It leverages the advantages of dual-band (infrared and visible light) imaging, considering semantic information, geometric structure, and local matching details within images to predict the disparity between heterogeneous image pairs accurately. CFNet demonstrates superior performance in recognized evaluation metrics and visual image observations when compared with other publicly available networks, offering an effective technical approach for practical heterogeneous image disparity prediction.

## 1. Introduction

Dual-band infrared–visible imaging technology is prevalent in the military sector, autonomous driving assistance systems [1], disaster relief robots [2], and small unmanned aerial vehicles (UAVs) [3]. It effectively facilitates visual tasks such as target identification [4], tracking [5], and scene enhancement [1,2,3]. Owing to constraints such as system volume, weight, and cost, a common configuration involves an infrared camera paired with a visible light camera to form a system for heterogeneous image acquisition. The visual tasks achieved through this image acquisition system typically utilize the two-dimensional information of the target scene, such as using visible light or infrared imaging during the day and only infrared imaging at night. Despite recent developments in visible light (low-light) and infrared dual-band fusion imaging technology that have enhanced the amount of information gathered from the target scene, the depth information obtained from the target scene is not yet sufficient. This limitation hampers the accurate and objective environmental perception of imaging systems [6]. Therefore, researching stereoscopic vision technology based on heterogeneous binocular information leverages the complementary nature of dual-band scene information to achieve target identification and tracking in complex environments. Furthermore, it simultaneously provides information regarding the relative spatial position, depth, and dimensions of the target scene [7].

## 2. Related Work

Previously, the feasibility of achieving binocular stereo vision using scene images from different spectral bands has been demonstrated. Visible light images exhibit rich color, texture, and edge details with high contrast, making them suitable for human observation and target discrimination. In contrast, infrared images reflect thermal radiation information from the observed environment [8,9] and possess strong smoke transmission capability while being less affected by lighting conditions. With the spread of research and application of deep learning in image processing, stereo-matching algorithms have evolved from traditional local, global, and semi-global optimization methods to deep learning-based stereo-matching algorithms [10]. Leveraging the complementary advantages of multiband sensors on existing heterogeneous imaging systems has become a significant research direction for binocular stereo-vision technology. Multispectral image stereo matching involves identifying corresponding feature points between heterogeneous image pairs to compute disparity values. Kim et al., in 2015, introduced the dense adaptive self-correlation (DASC) matching descriptor [11], which performed feature point matching on two spectral band images. In 2018, Zhi et al. proposed an unsupervised cross-spectral stereo-matching (CS-Stereo) method based on deep learning [12], consisting of disparity prediction and spectral transformation networks. An evaluation function for material perception was integrated into the disparity prediction network to handle unreliable matching regions such as light sources and glass. Liang et al. improved Zhi’s network structure in 2019 [13] by using a spectrally adversarial transformation network (F-cycleGAN) to enhance the quality of disparity prediction. In 2022, Liang et al. added a multispectral fusion subnetwork to the previous two network architectures [14], minimizing cross-spectral differences between visible light and near-infrared images through fusion. The aforementioned networks are more suitable for visible light–near-infrared image pairs with minor spectral differences; however, their performance is not ideal for visible light–thermal infrared image pairs with more significant spectral differences. In 2020, Li et al. proposed a depth prediction network called IVFuseNet, which extracts common features from infrared and visible light images [15]. However, it overlooks semantic image information, limiting its prediction accuracy.

In recent years, iterative networks have demonstrated promising performance in homogenous image stereo-matching tasks [16,17,18]. Lipson et al., in 2021, proposed RAFT-Stereo [16], which employs local loss values obtained from all-pair correlations to optimize and predict the disparity map iteratively. However, the capacity of this network for extracting and utilizing global information is insufficient and, thus, it struggles with local ambiguities in inappropriate regions. In 2023, Xu et al. [17] addressed the limitations of RAFT-Stereo by introducing the IGEV-Stereo network. This network constructs a structure through which to encode global geometry and contextual information, along with local matching details, enhancing the effectiveness of the iterative process. The IGEV-Stereo network was designed for the stereo matching of visible light image pairs. It processes input image pairs through a feature extraction subnetwork to obtain two feature maps from the left and right views. These maps are subjected to correlation calculations in order to generate a correlation volume, which is subsequently fed into a lightweight encoder–decoder structure to produce a geometry-encoding volume. This volume offers an improved initial disparity map for the iterative convolutional gated recurrent units (ConvGRUs), thus accelerating network updates. Furthermore, it incorporates global geometry and semantic information, enabling the network to better address local ambiguity issues in pathological regions.

In response to the limitations of existing methods for predicting disparities in heterogeneous image pairs, we propose an iterative network for disparity prediction with infrared and visible light images based on common features (CFNet). Building upon the extraction of common features, CFNet comprehensively considers the unique information from each heterogeneous image. It integrates global geometry, local matching, and individual semantic information from the heterogeneous images into a cascaded iterative optimization module. Furthermore, CFNet leverages the geometry-encoding volume produced with a three-dimensional (3D) regularization network, regresses it, and obtains an initial disparity value, thereby expediting convergence and reducing prediction errors.

The remainder of this article is structured as follows: Section 2 introduces the proposed method, detailing the structure and roles of various sub-modules within the network and the composition of the loss function. Section 3 compares our network’s experimental results with those of other methods and provides the outcomes of ablation experiments. Finally, Section 4 provides an overall evaluation of the network.

## 3. Methods

**Figure 1:**
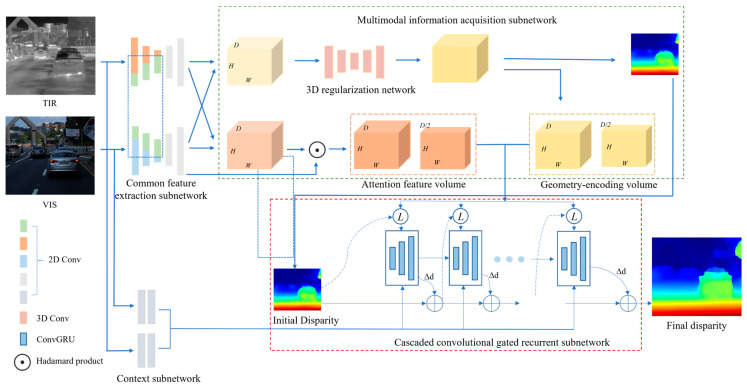
Overview of the proposed network.

The proposed CFNet architecture is shown in Figure 1. The input consists of heterogeneous infrared–visible image pairs, which are initially processed through a common feature extraction subnetwork for feature extraction. The green blocks within the blue dashed box represent the common features extracted from both infrared and visible light images. The context subnetwork extracts semantic features from the heterogeneous images, serving as the initial hidden state for the convolutional gated recurrent units (ConvGRUs). The green dashed box contains the multimodal information acquisition subnetwork, wherein a 3D regularization network generates a geometry-encoding volume, and an attention feature volume is obtained using the values of the correlation volume as attention weights. These two features are combined and passed to the next network level. Additionally, the geometry-encoding volume is utilized to derive an initial disparity map, which accelerates network updates. In the cascaded convolutional gated recurrent subnetwork within the red dashed box, each ConvGRU level receives the joint encoding from the common feature extraction subnetwork, the contextual information of the heterogeneous images from the context network, and the disparity update information from the previous ConvGRU level. After multiple ConvGRU computations are performed, the disparity values are updated.

### 3.1. Common Feature Extraction Subnetwork

Despite the distinct characteristics exhibited by infrared thermal radiation images $I_{l}\in {\mathbb{R}}^{1\times H\times W}$ and visible light images $I_{r}\in {\mathbb{R}}^{3\times H\times W}$—where *H* and *W* denote the length and width dimensions of the original image, and subscripts “*l*” and “*r*” designate the correspondence of the image to the left or right feature map groups, respectively—infrared images of various scenes contain the objects’ contour information due to variations in thermal radiation, while, in contrast, visible light images often exhibit edge contours owing to brightness or color differences. We refer to the similar features extracted from the same scene’s infrared–visible light image pair using coupled filters as “common features”, whereas the distinct differences displayed in their respective images, owing to spectral disparities, are termed “unique features”.

The common feature extraction subnetwork employs a dual-stream convolutional structure. In the downsampling stage, the filters in each layer are coupled, allowing for the extraction of common features from the infrared and visible light images. The filters used during the downsampling process in the common feature extraction subnetwork can be classified into three categories: filters for extracting unique features from the infrared image, filters for extracting unique features from the visible light image, and partially coupled filters for extracting common features from the heterogeneous image pair. Within this subnetwork’s dual-branch structure, the ratio of partially coupled filters to the total number of filters at the same sequential position in the convolutional layers is called the coupling ratio, denoted as *R_i_* and defined as

(1) $$R_{i}=k_{i}/n_{i}\;(i=1,2,3,4,5,6)$$

where, *R_i_* represents the coupling ratio of the *i*-th convolutional layer, *k_i_* denotes the number of partially coupled filters, and *n_i_* indicates the total number of filters.

Due to spectral differences, thermal infrared images and visible light images exhibit significant differences in detail, although both images contain “common features”. Shallow networks extract textural information from images, whereas deeper networks focus more on the structural and semantic information of objects. Therefore, the network design of this segment involved gradually increasing the coupling ratio with the deepening of convolutional layers. The coupling ratios used in this network were 0, 0.25, 0.25, 0.5, 0.5, and 0.75. Compared to IVFuseNet [15], which employs pooling layers for downsampling, our proposed network employs consecutive convolutional layers to simultaneously achieve downsampling and extract higher-level semantic information from feature maps, enhancing the network’s feature extraction and fusion capabilities. Additionally, multiple small-sized convolutional kernels are utilized to replace the large-sized kernels. This reduces the parameter count and enhances the acquisition of structural information from feature maps, thereby improving the model’s generalization ability. After consecutive downsampling, a feature map group with an original resolution of 1/32 is obtained. Subsequently, upsampling blocks with skip connections are employed to restore the sizes of the left and right feature map groups to 1/4 of the original resolution, resulting in a multiscale feature map group:

(2) $$\begin{array}{l} f_{l,i}\in {\mathbb{R}}^{C_{i}\times \frac{H}{i}\times \frac{W}{i}}\\ f_{r,i}\in {\mathbb{R}}^{C_{i}\times \frac{H}{i}\times \frac{W}{i}} \end{array}$$

**Figure 2:**
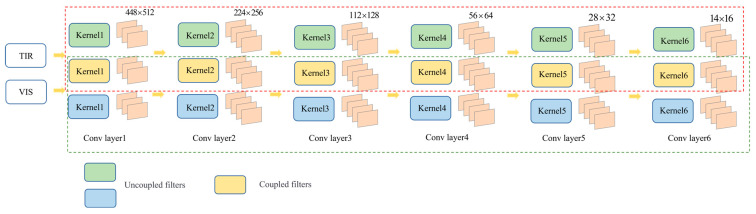
Downsampling process of the common feature extraction subnetwork for image pairs.

Here, *C_i_* represents the number of feature channels, while $f_{l,4}$ and $f_{r,4}$ are utilized to construct the cost volume. The network flow of the downsampling process in the common feature extraction subnetwork is depicted in Figure 2, and its primary structure is presented in Table 1. The red dashed box represents the processing flow for infrared images, whereas the green dashed box corresponds to that for visible light images. The overlapping portion between the two represents the extraction of common features from the image pair using coupled filters.

### 3.2. Context Subnetwork

**Figure 3:**
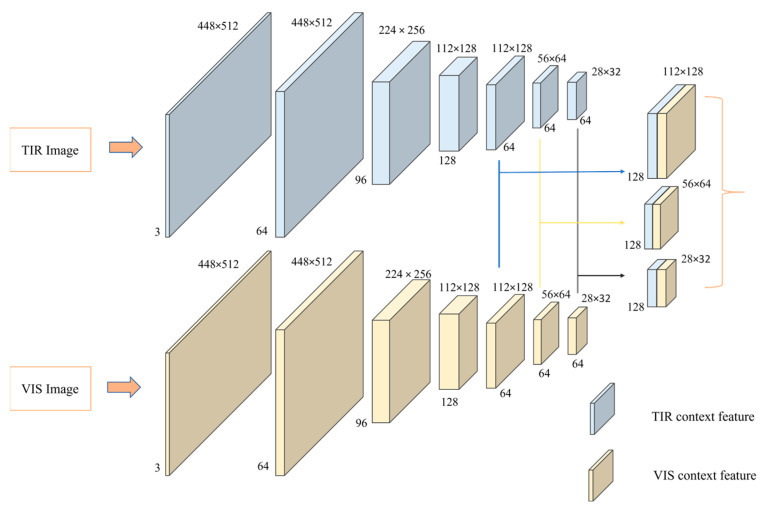
Evolutionary process of the feature map group in the context subnetwork.

The input to the network consists of heterogeneous image pairs representing the left and right views. Owing to significant spectral differences between the images, the left and right views contain distinct contextual information. Therefore, this network extracts contextual information separately for each view. The context network comprises two branches with identical structures, each with a residual module series. First, the network generates feature map groups for the left and right views at resolutions of 1/4, 1/8, and 1/16 of the input image, with each feature map group having 64 channels. These feature map groups capture contextual information at different scales. Subsequently, feature map groups of the same size generated from the left and right views are stacked together. Finally, the contextual information obtained at different scales is used to initialize and update the hidden states of ConvGRU, and the evolution of its feature map group is shown in Figure 3.

### 3.3. Multimodal Information Acquisition Subnetwork

Different processes were applied to the feature map groups extracted from the left and right views to obtain a more comprehensive geometric structure and local matching information from the heterogeneous image pair in the multimodal information acquisition subnetwork.

The extracted feature map groups from the left and right views constructed a correlation volume. These feature map groups, $f_{l,4}$ and $f_{r,4}$, were divided into *g* = 8 groups along the channel dimension, and the correlation mapping is computed for each group by

(3) $$C_{corr}(g,d,x,y)=\frac{1}{N_{c}/g}\langle f_{l,4}^{}(x,y),f_{r,4}^{}(x-d,y)\rangle$$

where *x* and *y* represent the pixel coordinates of feature points in the feature map; *d* is the disparity index, with values ranging from 0 to 192; *N_c_* denotes the number of feature channels; and $\langle \cdot ,\cdot \rangle$ indicates the inner product.

Since the cost volume $C_{corr}$, based on feature correlation, focuses solely on local geometric information, this does not facilitate the network’s utilization of global image information to achieve better stereo-matching results. Inspired by the CEGV structure of the IGEV-Stereo network [17], a 3D regularization network, denoted as *R*, was employed to further process the corresponding cost volume of the left feature map group $C_{corr}^{l}$. *R* is a lightweight encoder–decoder network; whereas the upsampling and downsampling modules consist of 3D convolutions, this network effectively extracts and propagates feature information from the feature map groups of different scales [19], resulting in an encoded volume $C_{G}$ that combines global geometry and semantic information. The generation process is as follows:

(4) $$C_{G}=R(C_{corr}^{l})$$

Then, the corresponding cost volume of the right feature map group $C_{corr}^{r}$ was further encoded for matching and semantic information through the construction of an attention feature volume [20,21]. This was primarily due to the significant spectral differences between the input heterogeneous image pair, where different views contain more distinct semantic information. Using the cost volume values as attention weights efficiently enhances the extraction of image features.

The construction of the attention feature volume initially involves adjusting the channel count of the cost volume $C_{corr}^{r}$ using a 3 × 3 convolution operation to obtain a weight matrix, $A_{corr}$. Subsequently, two consecutive 1 × 1 convolution operations are applied to adjust the channel count of the right feature map group $f_{r,4}$ to 8, followed by activation using the sigmoid function to generate the adjustment matrix $F_{r}$. Finally, the attention feature volume $V_{AF}$ is computed as

(5) $$V_{AF}=A_{corr}⊙{\;F}_{r}$$

where ⊙ represents the Hadamard product, indicating element-wise multiplication between two matrices.

We further downsampled $C_{G}$ and $V_{AF}$ to obtain two pyramid-structured feature map groups of the same size. Stacking these two pyramid-structured feature map groups at corresponding positions results in a new pyramid-level-structured feature map group called the joint encoding volume, $C_{v}$.

### 3.4. Cascaded Convolutional Gated Recurrent Subnetwork

Deep feature maps contain more semantic information and larger receptive fields, making networks more robust in stereo matching within non-textured or repetitively textured regions. However, these feature maps may require more fine structural details. To strike a balance between network robustness and the perceptual ability for image details [17], the network also employs the ConvGRU structure for the iterative optimization of disparity values.

The initial disparity map *d*_0_ is first computed from the geometry-encoding volume (*C_G_*) using the soft-argmin method, where

(6) $$d_{0}=\sum_{d=0}^{D-1}d\times \mathrm{softmax}(C_{G}(d))$$

**Figure 4:**
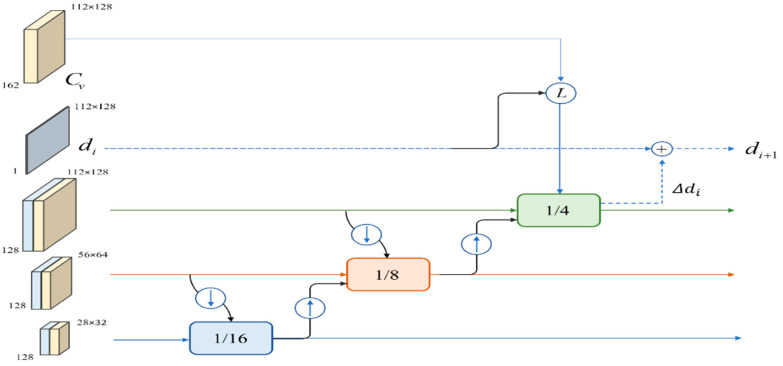
Multilevel ConvGRU.

Starting from *d*_0_, the ConvGRU modules are utilized for iterative disparity map updates to aid in rapid convergence optimization of the disparity computation. Each level of ConvGRU accepts the joint encoding volume $C_{v}$, the semantic features extracted with the context subnetwork, and the disparity update information passed from the previous ConvGRU level. As shown in Figure 4, from employing a 3-level ConvGRU, feature maps with sizes corresponding to 1/16, 1/8, and 1/4 of the original input image are processed. The information within the feature maps is connected using pooling and upsampling operations, and the outputs of the previous ConvGRU levels are cascaded as input hidden states to the subsequent ConvGRU level. Ultimately, the disparity map is updated using the output from the final level (denoted in green) ConvGRU.

After the computations through the multilevel ConvGRU, the updated disparity map $\Delta d_{i}$ is obtained for updating the current disparity value *d_i_* as follows:

(7) $$d_{i+1}=d_{i}+\Delta d_{i}\;\left(i=0,1,2\ldots N-1\right)$$

### 3.5. Loss Function

The computation of the loss value can be divided into two parts. Using the initial disparity map *d*_0_ and all disparity prediction results, ${\left\{d_{i}\right\}}_{i=1}^{N}$, obtained after each iteration of ConvGRU to calculate *L*_1_ loss, the final expression for the loss function is as follows:

(8) $$L_{stereo}={\mathrm{Smooth}}_{L_{1}}(d_{0}-d_{gt})+\sum_{i=1}^{N}\gamma^{N-i}{\left\|d_{i}-d_{gt}\right\|}_{1}$$

where *d*_0_ represents the initial disparity map; *d_gt_* represents the transformation of distance information acquired using a LiDAR sensor into a corresponding disparity map aligned with the left view, set as the ground truth map in this study; γ is set to 0.9 within the network, and the number of forward passes for disparity updates was is to 22; and ${\mathrm{Smooth}}_{L_{1}}$ serves as a smoothing loss function and is calculated as follows:

(9) $$Smooth_{L_{1}}(d_{0}-d_{gt})=\left\{\begin{array}{ccc} 0.5{\left(d_{0}-d_{gt}\right)}^{2} & & \left|d_{0}-d_{gt}\right|<1\\ \left|d_{0}-d_{gt}\right|-0.5 & & otherwise \end{array}\right.$$

## 4. Experimental Procedure

### 4.1. Experimental Dataset and Evaluation Benchmark

**Figure 5:**
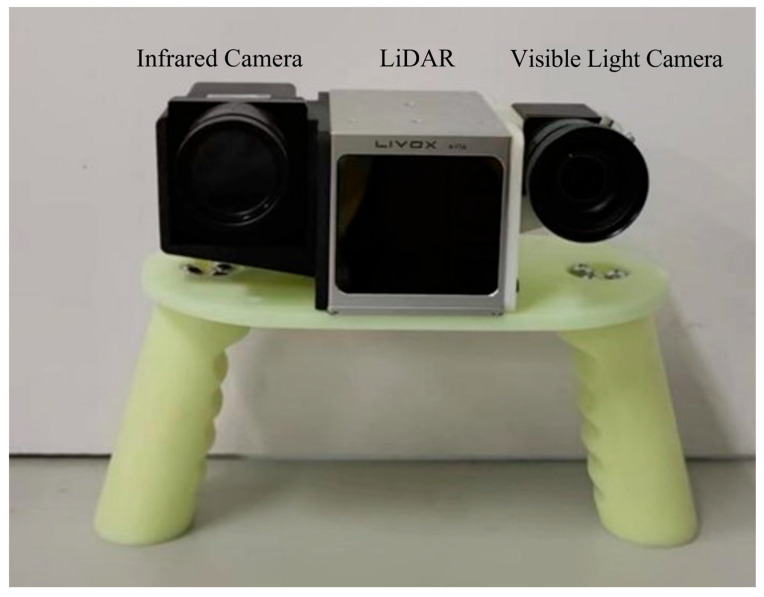
Structural design of the data acquisition equipment.

The experimental data consist of two parts: the publicly available multispectral dataset, KAIST [22], and the data obtained from a self-designed and constructed data acquisition system, which contains thermal infrared–visible light image pairs and corresponding disparity maps. The KAIST multispectral dataset [22] encompasses heterogeneous images captured in various settings such as road scenes in urban and rural areas, covering both daytime and evening scenarios, with the scenes mainly including vehicles, pedestrians, and traffic signs. The primary reason for selecting the KAIST dataset was its inclusion of stereo-rectified pairs of thermal infrared and visible light images, along with distance information obtained through a LiDAR sensor for objects in the scene. The self-designed data acquisition system in this study consists of a fixed arrangement with a visible light camera, a thermal infrared camera, and a LiDAR, as shown in Figure 5. On the left is the thermal infrared camera, in the middle is the LiDAR, and on the right is the visible light camera. The specific parameters of the equipment are detailed in Table 2 and Table 3. The two cameras captured the left and right views. The Livox Avia LiDAR was employed to acquire distance information, then, the distance information was converted to the ground truth disparity map based on the baseline length and focal length obtained from the stereo calibration of the camera system. The ground truth disparity map was subsequently used to calculate the loss with the predicted *d_i_* as well as initial and final disparity maps obtained from the network. The data collection primarily focused on on-campus environments and mainly included parked vehicles and people in a stationary state. Approximately 300 sets of data were collected in total, and the dataset was divided into training and test sets in an 8:2 ratio.

To train and evaluate the stereo disparity prediction methods, we divided the KAIST dataset [22] into training and testing sets in a ratio of 29:1. The training set was used for training the network, whereas the testing set was employed to predict disparities using various network models. Widely recognized evaluation metrics [23] were then utilized to assess the performance of different stereo disparity prediction methods objectively. The specific evaluation metrics and their computation methods are presented in Table 4. As shown, represents the ground truth disparity value, and denotes the predicted disparity value; the units for both are pixels. The root mean square error (RMSE), logarithmically scaled RMSE (log10 RMSE), absolute relative difference (Abs Rel), and squared relative difference (Sq Rel) were used to quantify differences between the predicted and ground truth values. A lower computed value indicates a higher prediction accuracy of the network. Threshold accuracy measures the similarity between the predicted and ground truth disparities, with higher computed values indicating better network prediction accuracy.

### 4.2. Implementation Details

**Figure 6:**
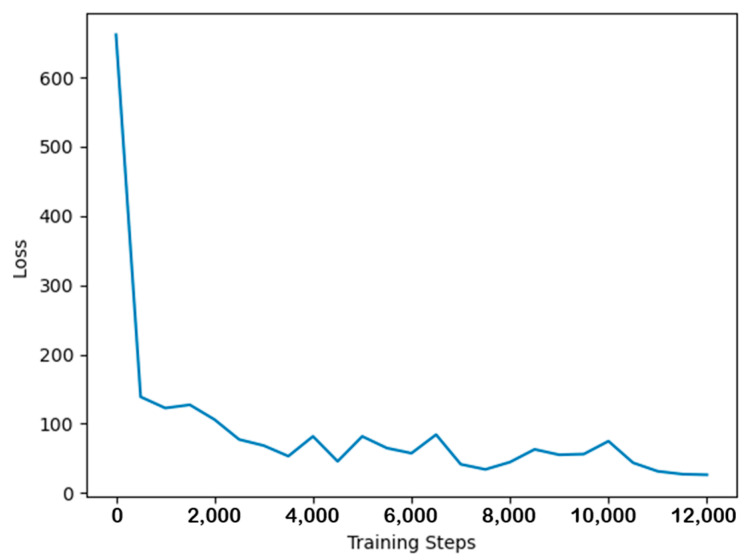
Loss value variation curve.

**Figure 7:**
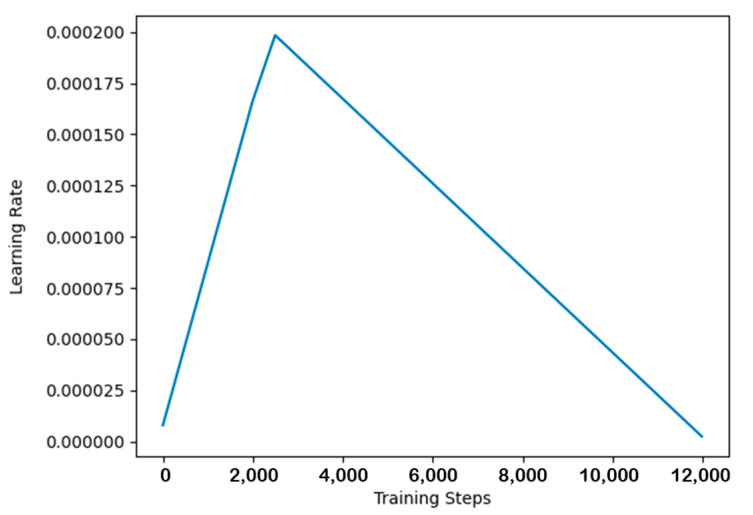
Learning rate variation curve.

The network was trained using the KAIST dataset [22], a batch size of 4, and 12,000 steps. The entire training process was conducted on two NVIDIA RTX 3090 GPUs. The variation in loss values during the training process is depicted in Figure 6. During network training, the gradients were adjusted to the range of [−1, 1]. The AdamW optimizer was employed to update the network parameters, and a one-cycle learning rate adjustment strategy was used, with a maximum learning rate of 0.0002. The variation in the learning rate is shown in Figure 7.

### 4.3. Experimental Results

**Figure 8:**
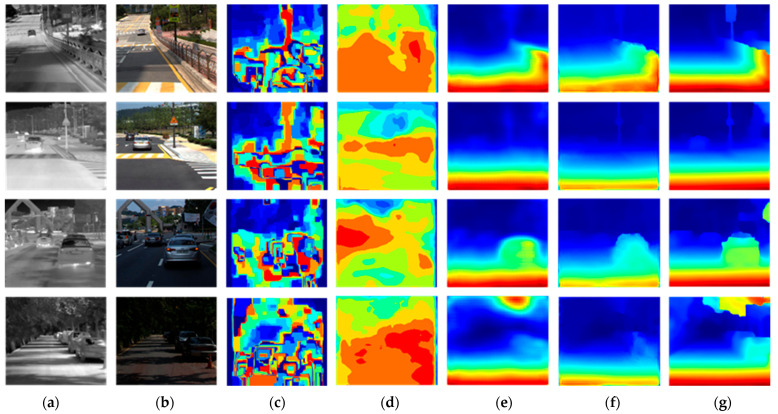
Comparison of experimental results using the testing dataset: (**a**) thermal infrared image, (**b**) visible light image, (**c**) DASC, (**d**) CS-Stereo, (**e**) IVFuseNet, (**f**) CREStereo, and (**g**) our network.

**Figure 9:**
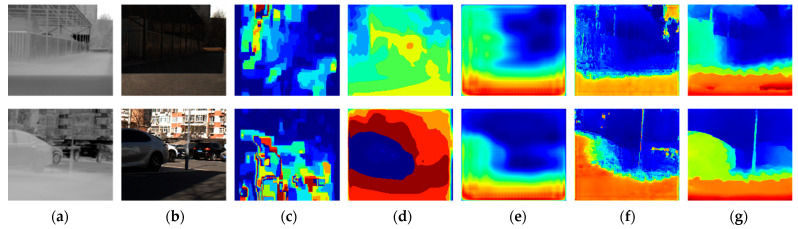
Comparison of experimental results using the custom dataset: (**a**) thermal infrared image, (**b**) visible light image, (**c**) DASC, (**d**) CS-Stereo, (**e**) IVFuseNet, (**f**) CREStereo, and (**g**) our network.

The performance of CFNet was compared with other publicly available networks such as DASC [11], CS-Stereo [12], IVFN [15], and CREStereo [18]. For networks initially designed for infrared–visible light stereo matching, pre-trained weight files were directly loaded into the network for testing. Networks that were initially designed for visible light stereo matching were trained on the same dataset before being evaluated. We evaluated the prediction results of various methods using similar metrics to compare the networks. Table 5 presents detailed evaluation and prediction results. Figure 8 shows the disparity prediction results obtained from a subset of heterogeneous image pairs in the testing dataset [22]. Figure 9 displays the disparity prediction results obtained from a subset of heterogeneous image pairs in the self-collected dataset.

DASC, a traditional method [11], can be directly applied to heterogeneous image stereo matching without extensive training. However, its generalization to different scenes is limited due to its manual feature extraction, which only utilizes partial image information. Moreover, setting empirical parameters significantly affects its matching performance, leading to suboptimal results. The CS-Stereo network [12] combines spectral translation and disparity prediction networks. To predict disparities, cross-spectral translation of two different spectral images is required. The network generates pseudo-images from real images and calculates disparities for both, with the main error originating from pixel-level translation inaccuracies. Given the substantial spectral differences between thermal infrared and visible light heterogeneous image pairs, achieving accurate cross-spectral translation poses significant challenges, resulting in unsatisfactory prediction performance. IVFuseNet [15] uses a dedicated subnetwork to extract common features from heterogeneous image pairs. This design captures both common and unique features of the heterogeneous images, effectively utilizing their complementary information. However, IVFuseNet does not fully exploit semantic information from images, leading to suboptimal performance in regions with large texture-less areas or repetitive structures. Regarding the CREStereo network [18], its iterative optimization approach aids in generating high-resolution disparity maps. However, it lacks the utilization of complementary information in heterogeneous image pairs. Additionally, when using the ConvGRU module, setting the initial value of disparity to 0 reduces the optimization efficiency of the network during the training process.

CFNet achieves the best evaluation metrics compared to publicly available networks, with the lowest values for RMSE, Log10 RMSE, Abs Rel, and Sq Rel. These results indicate that the disparities predicted with the network are closest to the ground truth disparities. Furthermore, examining its accuracy across different thresholds reveals that deviations between predicted and ground truth disparities are consistently controlled within a certain range throughout the image. This demonstrates the robustness of our method in predicting disparities across various environmental conditions and providing reliable depth estimations for objects in the image. We employed multilevel partial-coupling filters in the common feature extraction subnetwork to leverage the complementary advantages of thermal infrared and visible light images. This approach enabled the partial common features in both images to be treated as auxiliary variables in the feature extraction process. Additionally, we optimized the inputs of the network’s iterative modules to balance global structural information, local matching details, and semantic information from both heterogeneous images. This enhancement contributed to the effectiveness of disparity updates in each iteration module. As shown in Figure 8, our proposed network accurately predicts disparities for large objects on the road, such as vehicles at different distances. The contours of vehicles in the disparity map are more distinct and complete. The network successfully identifies and predicts the posts of traffic signs and, even in the presence of repetitive textural information on road barriers, achieves robust matching results, clearly distinguishing different barrier distances in the disparity map. Furthermore, as shown in Figure 9, the network demonstrates good generalization performance in various environments. After transfer training using our custom dataset, the network accurately predicted disparities for closely spaced vehicles in a parking lot and accurately represented them in the disparity map. The network can also reliably match continuous fences and lamp posts, yielding clear indications in the disparity map. Multiple experimental results substantiate that our proposed network achieves high-quality stereo matching for heterogeneous image pairs, and its visualized results surpass those of other methods.

### 4.4. Ablation Experiments

We conducted ablation experiments to evaluate the impact of each module on the generalization ability and prediction accuracy of the proposed network. We used the IGEV-Stereo network as the baseline. As presented in Table 6, we compared the performance of the baseline network with that of the network improved by addition of the common feature extraction network or the multimodal information acquisition network to validate the effectiveness of each submodule. After partially coupled filters were used in the feature extraction module, the Abs Rel error between the predicted results and ground truth values decreased. This is primarily because we leveraged the complementarity of the heterogeneous image pair and used visible light and thermal infrared images as auxiliary variables for feature extraction from each other. When processing the feature maps extracted from the left and right views, semantic information was not solely extracted from the left feature maps. Instead, we used different modules to extract the global geometric information, local matching information, and their respective semantic information separately from different feature maps. This enabled the network to obtain comprehensive information from heterogeneous image pairs. Integrating this information and feeding it into the subsequent iterative modules improved the prediction accuracy of the network.

## 5. Conclusions

In addressing the challenge of stereo matching for heterogeneous infrared–visible image pairs, this study presented CFNet, an iterative network for predicting disparity with infrared and visible light images based on common features. Compared to other networks, the CFNet integrates a common feature extraction subnetwork with cascaded convolutional gated recurrent subnetwork, which enables the network to effectively harness the complementary advantages of both spectral domains, incorporating semantic information, geometric structure, and local matching details in the images. This results in more accurate disparity predictions for scenes within heterogeneous image pairs. Existing methods have not exploited the complementary information in heterogeneous images or have not effectively utilized the semantic information from images. Additionally, an initial disparity value of 0 leads to use of more training iterations, which reduces the optimization efficiency. The disparity prediction performance of CFNet surpassed those of other methods, as evidenced by the superior results in recognized evaluation metrics (RMSE, log10 RMSE, Abs Rel, and Sq Rel). Visualizing the predicted disparity maps further demonstrated the superiority of CFNet compared to other publicly available networks.

Currently, parallel optical paths in multispectral imaging systems have extensive applications, often in switching or fusion imaging modes. However, these systems do not effectively utilize their field of view to acquire disparity information. CFNet directly leverages the heterogenous infrared–visible image pairs for stereo matching, enabling the system to perceive the disparity information from the image pairs without additional sensors. This approach enhances the system’s ability to perceive the surrounding environment while avoiding hardware complexity. Consequently, the system’s overall structure becomes more conducive to integrated design and precise calibration, facilitating the broader adoption of heterogeneous image acquisition systems.

## Figures and Tables

**Table 1:** Structure of the common feature extraction in the downsampling process.

Layers	Kernel	Strides	Channels	Output Size	Couple Ratio
Conv1	3 × 3	1	16	448 × 512	0
Conv2	3 × 3	2	32	224 × 256	0.25
Conv3	3 × 3	2	48	112 × 128	0.25
Conv4	3 × 3	2	64	56 × 64	0.5
Conv5	3 × 3	2	192	28 × 32	0.5
Conv6	3 × 3	2	160	14 × 16	0.75

**Table 2:** Specific parameters of the cameras.

Camera Type	Pixel Size	Resolution	Focal Length	Field of View
Visible Light Camera	2.4 μm	3072 × 2048	6 mm	63.1° × 44.5°
Infrared Camera	17 μm	640 × 512	25 mm	24.6° × 19.8°

**Table 3:** Specific parameters of the LiDAR.

Scanning FOV	Maximum Detection Range	Accuracy	Laser Wavelength
70.4° × 77.2°	450 m	±20 mm	905 nm

**Table 4:** Evaluation metrics and calculation methods.

Evaluation Metric	Calculation Method
Root mean square error (RMSE)	$\sqrt{\frac{1}{N}{\sum_{i}^{N}\left\|y_{i}-y_{i}^{*}\right\|}^{2}}$
RMSE of log_10_ (log_10_ RMSE)	$\sqrt{\frac{1}{N}\sum_{i}^{N}{\left\|\log_{10}y_{i}-\log_{10}y_{i}^{*}\right\|}^{2}}$
Absolute relative difference	$\frac{1}{N}\sum_{i}^{N}\left\|y_{i}-y_{i}^{*}\right\|/y_{i}^{*}$
Squared relative difference	$\frac{1}{N}{\sum_{i}^{N}\left\|y_{i}-y_{i}^{*}\right\|}^{2}/y_{i}^{*}$
Threshold: % of $y_{i}$ s.t.	$\max(\frac{y_{i}}{y_{i}^{*}},\frac{y_{i}^{*}}{y_{i}})=\delta <thr$

**Table 5:** The present study’s results: CFNet compared with other methods.

Method	RMSE	Log RMSE	Abs Rel	Sq Rel	$\delta <1.25^{1}$	$\delta <1.25^{2}$	$\delta <1.25^{3}$
DASC	9.8257	0.3981	1.3618	14.5404	0.0981	0.1906	0.2941
CS-Stereo	5.5906	0.3387	1.1428	8.6146	0.2587	0.4212	0.5715
IVFN	1.3857	0.0959	0.1593	0.2516	0.5589	0.7438	0.8541
CREStereo	1.4663	0.1285	0.1835	0.3584	0.6355	0.8373	0.9414
**CFNet**	**1.3743**	**0.0913**	**0.1432**	**0.2314**	**0.8597**	**0.9359**	**0.9731**

**Table 6:** Ablation study of the proposed networks.

	Baseline Model	Baseline + Common Feature Extraction Subnetwork	Baseline + Multimodal Information Acquisition Subnetwork	Complete Model
RMSE	1.4223	1.4027	1.3801	**1.3743**
Abs Rel	0.1588	0.1537	0.1520	**0.1432**
$\delta <1.25^{1}$	0.8331	0.8406	0.8515	**0.8597**
$\delta <1.25^{3}$	0.9678	0.9727	0.9728	**0.9731**

## Data Availability

We used three public datasets: KAIST: https://github.com/SoonminHwang/rgbt-ped-detection (accessed on 18 May 2023).

## Abbreviations

FOV	Field of view
RMSE	Root mean square error
Log RMSE	RMSE of log10
Abs Rel	Absolute relative difference
Sq Rel	Squared relative difference
